# Advances in peptides encoded by non-coding RNAs: A cargo in exosome

**DOI:** 10.3389/fonc.2022.1081997

**Published:** 2022-12-23

**Authors:** Jing Yang, Mengxiao Liu, Xidong Fang, Huiyun Zhang, Qian Ren, Ya Zheng, Yuping Wang, Yongning Zhou

**Affiliations:** ^1^ The First Clinical Medical College, Lanzhou University, Lanzhou, China; ^2^ Department of Gastroenterology, The First Hospital of Lanzhou University, Lanzhou, China; ^3^ Key Laboratory for Gastrointestinal Diseases of Gansu Province, The First Hospital of Lanzhou University, Lanzhou, China

**Keywords:** non-coding RNA, exosome, peptide, translation, cancer

## Abstract

The metastasis of malignant tumors determines patient prognosis. This is the main reason for the poor prognosis of patients with cancer and the most challenging aspect of treating malignant tumors. Therefore, it is important to identify early tumor markers and molecules that can predict patient prognosis. However, there are currently no molecular markers with good clinical accuracy and specificity. Many non-coding RNA (ncRNAs)have been identified, which can regulate the process of tumor development at multiple levels. Interestingly, some ncRNAs are translated to produce functional peptides. Exosomes act as signal carriers, are encapsulated in nucleic acids and proteins, and play a messenger role in cell-to-cell communication. Recent studies have identified exosome peptides with potential diagnostic roles. This review aims to provide a theoretical basis for ncRNA-encoded peptides or proteins transported by exosomes and ultimately to provide ideas for further development of new diagnostic and prognostic cancer markers.

## 1 Introduction

According to the statistical results of the World Health Organization in 2019, cancer is the first or second leading cause of death before the age of 70 years in 112 of 183 countries. Furthermore, the status of cancer as a leading cause of death continues to rise (1). During the growth and development of the body, oncogenes and tumor suppressor genes accumulate mutations due to repeated stimulation by various factors *in vivo* and *in vitro*, thus triggering the process of tumor development and metastasis, most of which are located in genomic regions lacking protein-coding capacity (2). However, currently available tumor biomarkers and therapeutic targets have been identified based on coding genes. Genome-wide association studies have identified many genetic variants associated with tumors and other diseases, two-thirds of which are located in non-coding regions (3, 4).

Non-coding RNAs (ncRNAs) is RNA molecules transcribed by the genome that do not encode a protein. NcRNAs can be divided into two main types: basic structural ncRNA and regulatory ncRNA. Basic structural ncRNAs are composed of ribosomal RNAs (rRNAs), transfer RNAs (tRNAs) and small nuclear RNAs (snRNAs). The functions of these ncRNAs are relatively well defined. Regulatory ncRNA including three main types: microRNAs (miRNAs), long non-coding RNAs (lncRNAs), and circular RNAs (circRNAs) (5, 6). Initially, ncRNAs were considered non-functional byproducts of RNA polymerase II transcripts and existed as “transcriptional noise” in the genome (7). However, there is growing evidence that ncRNAs have various essential functions in cancer cell growth, development, apoptosis, and metabolism (8–11) (**Figure S1**). The functions of the ncRNA molecules described above are based on their RNA molecules. Recent research on ncRNA molecules in tumors, such as breast, liver, and intestinal cancers has shown that ncRNAs can encode translation to produce functional peptides or protein molecules that participate in the primary developmental processes of various organisms and regulate the development of tumor cells (12–14). Although the functions of some peptides have been demonstrated, the mechanism of their action in cancer remains unclear. Studies have shown that exosomes are essential carriers of intercellular communication and contain a variety of functional molecules, such as mRNA, miRNAs, lncRNAs, circRNAs, and proteins (15–17). Exosome membranes influence the biological behavior of target cells by delivering their contents to them, thus influencing the biological behavior of the latter.

Interestingly, exosomes and peptides may also work together to deliver signaling molecules to modify or modulate receptor cells. This review summarizes the current studies concerning the relationship between exosomes and ncRNA with encoding potential in cancers. This review aimed to provide a theoretical basis for the study of the mechanisms of peptides encoded by ncRNAs.

## 2 Prediction and validation of novel peptides encoded by ncRNAs

A small percentage of ncRNAs contain a small open reading frame (smORF, < 300 nucleo tide) that can be translated into a peptide. Translation of ncRNAs has been neglected mainly because the peptides or proteins are encoded in a noncanonical mode, and the peptides are unstable and rapidly degraded (18, 19). However, owing to advances in sequencing technology, many studies have found that ncRNAs encode peptides and proteins during cancer development (20). To identify hidden peptides in ncRNAs, there are tools to identify smORFs with coding potential, such as CPC2 (21), ORF finder (22), LGC (23), ORF-RATER (24), CPAT (25), ORFscore (26), PhyloCSF (27), and PLEK (28). In the input box on the CPC2 homepage, you can simply paste or upload the FASTA format of the nucleotide sequences to obtain a report containing coding probability, putative peptide length, Fickett score, and isoelectric point (21). We then entered the DNA sequence in the ORF finder search box to search for potential protein-coding fragments and used SMART BLAST or BLASTP to validate the predicted proteins (22). LGC was the first new algorithm based on the difference in ORF length and GC content between lncRNAs and protein-coding RNAs. LGC outperforms the existing algorithms owing to its higher accuracy, sensitivity, and specificity (23). Based on these online tools (**Table 1**), we can initially predict whether a ncRNA of interest has coding properties. Using these tools, we will find some ncRNAs with encoding potential, and in the next step, we need to use some methods to verify the presence of these peptides. Mass spectrometry (MS) methods and peptidomics testing of encoded peptides are comparative direct evidence for the translability of ORFs. Build a fusion epitope tags, such as FLAG, GFP, HIS, etc., together with peptides/proteins to use western blot (WB), immunofluorescence to detect the presence of fusion proteins, and antibodies against peptide/protein can also be prepared for detection. CRISPR(clustered regularly interspaced short palindromic repeats)-Cas9 (CRISPR-associated protein 9) can insert the epitope tag into the site of the peptide/protein, and then use WB to detect the presence of the peptide/protein. Through the above experimental methods, we can confirm that the ncRNA can be translated into a peptide.

**Table 1:** Databases for ncRNAs prediction of coding peptides.

Database	Functions	Website	Reference
ORF finder	Search the potential protein encoding segments, verify predicted protein us SMART BLAST	https://www.ncbi.nlm.nih.gov/orffinder/	(22)
CPC2	Including coding probability, fickett score, isoelectric point	http://cpc2.gao-lab.org/index.php	(21)
CPAT	Including open reading frame size, open reading frame coverage, Fickett TESTCODE statistic, hexamer usage bias	http://lilab.research.bcm.edu/	(25)
CNCI	Coding label, score, length	http://www.bioinfo.org/software/cnci	(29)
PLEK	Coding label, coding potential score	https://sourceforge.net/projects/plek/	(28)
PhyloCSF	PhyloCSF score	http://compbio.mit.edu/PhyloCSF	(27)
LGC	ORF length, coding potential score, coding label	http://bigd.big.ac.cn/lgc/calculator	(23)
circRNAdb	Possible transcripts, IRES elements, open reading frame	http://reprod.njmu.edu.cn/circrnadb	(30)
IRES finder	Identification of IRES on RNA sequences	https://github.com/xiaofengsong/IRESfinder.	(31)
CircCode	Screening for circRNAs that bind ribosomes	https://github.com/PSSUN/CircCode	(32)
IRESite	Predicting whether circRNA has IRES sites	http://iresite.org/IRESite_web.php	(33)
IRESbase	Experimentally validated IRESs in the published literature were collected	http://reprod.njmu.edu.cn/cgi-bin/iresbase/	(34)
CircAtlas	Search ORFs of circRNA, Predicting the IRES in circRNA	http://circatlas.biols.ac.cn/	(35)

## 3 Functional ncRNAs-encoded peptides

### 3.1 MicroRNAs

**Figure 1:**
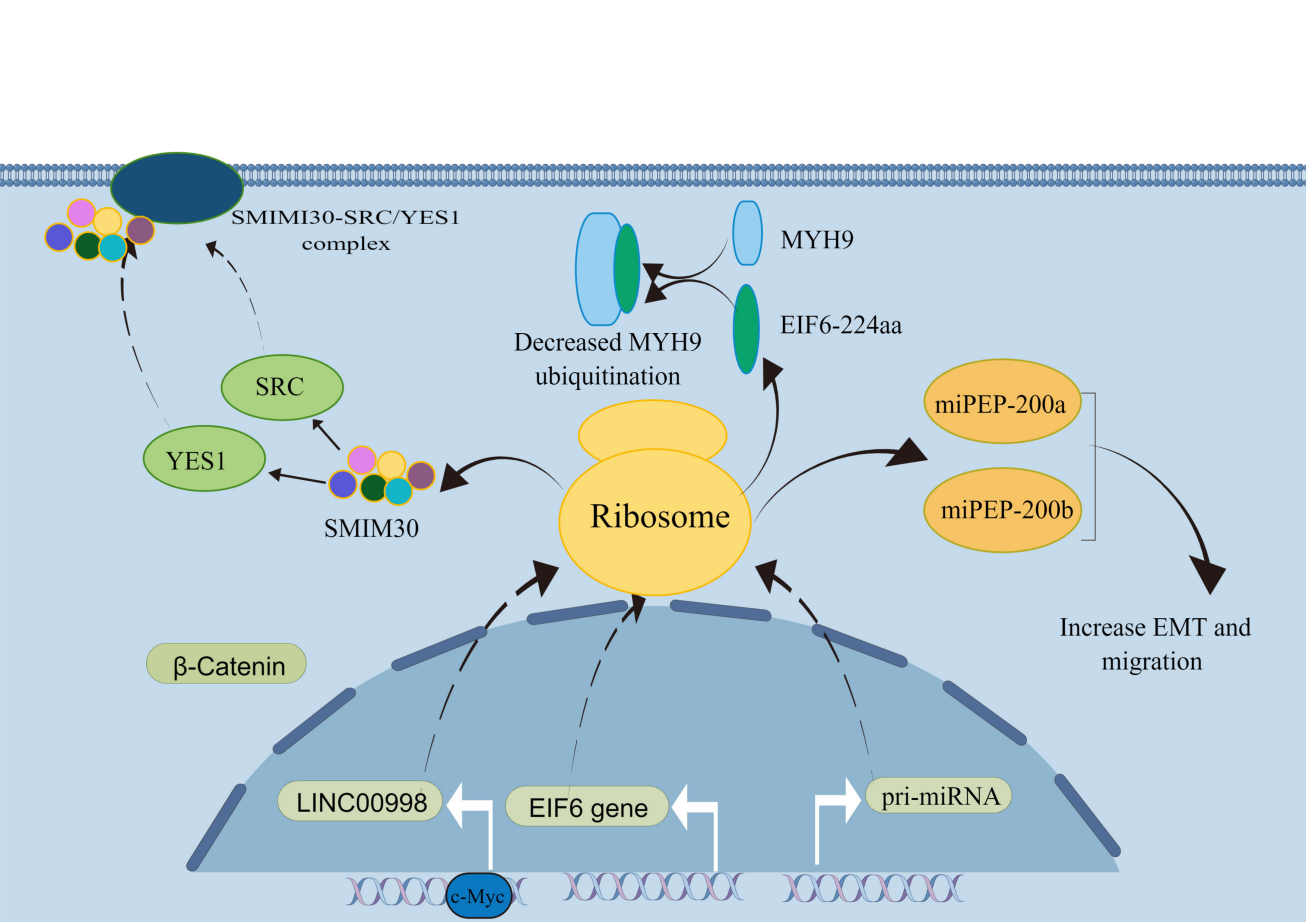
Role of ncRNAs-encoded peptides/protein in cancers. (By Figdraw).

MiRNAs are small RNAs approximately 20-25 nucleotides in length that regulate gene expression by explicitly cleaving or inhibiting the translation of target mRNAs (36, 37). The biogenesis of miRNAs undergoes two main steps. MiRNAs are first transcribed into primary transcripts of miRNAs (pri-miRNAs) in the nucleus. Then, miRNA is further processed to form a pre-miRNA for translocation into the cytoplasm and finally sheared by the Dicer enzyme. The mature miRNA binds to the 3’-UTR of the target mRNA to function (37). Usually, after a mature miRNA undergoes processing and excitement, the upstream and downstream sequences of the pri-miRNA become nonfunctional. They do not appear to be translated further to produce peptides or protein molecules (38). However, the current study shows that miRNA-encoded peptides (miPEPs) begin a new phase of gene regulation. Lauressegues et al. (36) found that in Arabidopsis, pri-miR171b and pri-miR165a have the potential to encode the peptides miPEP171b and miPEP165a, which downregulate downstream genes involved in root development. More importantly, Zhou et al. (39) found that pri-miRNA-31 encodes a peptide, miPEP31. MiPEP31 inhibits the transcription of miRNA-31 and downregulates its expression. Additionally, miRNA-31 is a negative regulator of Tregs-cells. MiPEP31 significantly inhibited experimental autoimmune encephalomyelitis by promoting Treg cell differentiation. This study found the peptide encoded by pri-miR-31 provides new approaches for the treatment of autoimmune disease. Current research on miRNAs is unclear as to whether miPEPs exist in all organisms and whether they all have a biological role. However, the relationship between miPEPs and tumors requires further investigation (**Figure 1**).

### 3.2 LncRNA

LncRNAs are a class of RNA molecules with transcripts longer than 200 nt transcribed by RNA polymerase II. It has a specific spatial secondary structure and a low interspecific conservation. LncRNA was initially considered as a redundant product of transcription. However, current studies suggest that it can undergo post-transcription modification, such as capping, polyadenylation, and splicing (40–42). Meanwhile, LncRNA generally does not encode proteins, but the number of molecules is much larger than protein-coding genes. LncRNA molecules are usually resided at separate locations on the genome and do not overlap with classical protein-coding genes, with some having small open reading frames (smORFs) (43). Using bioinformatics analysis, Pang et al. (12) identified LINC00998 as having a translational potential. The expression of peptide molecules corresponding to the smORF sequences was identified *in vitro* and *in vivo* by constructing fusion plasmids with FLAG tags. A recombinant plasmid fused to GFP was constructed, and SMIM30 was found to be localized to the cell membrane with a significant ability to guide membrane localization. In addition, extensive cellular functional assays revealed that the peptide SMIM30 plays a vital role in promoting the proliferation, invasion, and migration of hepatocellular carcinoma cells. Therefore, peptide SMIM30 may be used as a new prognostic indicator of hepatocellular carcinoma (HCC) and can provide a reference for the treatment of HCC patients. Guo et al. (13) identified LINC00665 in breast cancer that was significantly downregulated in translation after TGF-β treatment, further demonstrating that TGF-β treatment activates the Smad signaling pathway. Smad4 directly induced the expression of the translation repressor protein 4E-BP1, which led to a decrease in the expression of the peptide encoded by LINC00665 through direct binding to the eukaryotic translation initiation factor eIF4E. TGF-β acts as an oncogenic factor in advanced tumors and, to some extent, TGF-β induces metastasis and invasion in triple-negative breast cancer by regulating CIP2A-BP translation. In conclusion, the micropeptide protein CIP2A-BP, encoded by LINC00665, may soon become a potential therapeutic agent for treating triple-negative breast cancer metastasis. Although a limited number of peptides/proteins have been functionally characterized, their mechanisms of action in tumors remain unclear. Therefore, the study of ncRNAs molecules, especially lncRNAs, should not be confined to the RNA level but should also focus on whether they can be translated to produce functional protein molecules that can play a role in regulating basic life activities and disease development.

### 3.3 CircRNA

CircRNAs are produced from the exon or intron of a precursor messenger RNA by reverse splicing. CircRNAs are immune to digestion and degradation by nucleic acid exonucleases due to the absence of a 5’ end cap structure and a 3’ end tail (44). Currently, most circRNAs act as molecular sponges for miRNAs as the primary mechanism of action. However, in recent years, with the development of mass spectrometry and ribosome mapping, proteins or peptides produced by translation have become involved in the process of tumor development. There are two primary forms of protein translation: classical cap-dependent and non-classical non-cap-dependent. The non-cap mechanism allows cells to respond to a variety of transient stresses (45), and this translation is the mechanism underlying circRNA translation. In 2018, Yang et al. (46) discovered that circRNAs can encode peptides. In gliomas, circ-FBXW7 can encode a peptide FBXW7-185aa of 185 amino acids, which promotes the degradation of the proto-oncogene c-Myc by competitively binding USP28 ubiquitinated proteins, thereby inhibiting the development and progression of malignant gliomas.

Furthermore, high expression of circPPP1R12A was found in gastric cancer, encoding the production of the functional protein circPPP1R12A-73aa. In addition, cellular functional assays confirmed that it was the peptide but not circPPP1R12A that promoted the proliferation, invasion, and migration of gastric cancer cells *in vivo* and *in vitro* (47). Whether circRNAs that act through an endogenous competitive RNA mechanism can also act through peptides is interesting. A growing number of studies have confirmed that circRNA-encoded proteins or peptides have different mechanisms of action in tumors. Circ-EIF6 encodes EIF6-224aa which acts as a prognostic and therapeutic target in triple-negative breast cancer. This also suggests that circRNAs or the peptides they encode can be used as tumor markers and potential therapeutic targets (**Table 2**).

**Table 2:** ncRNAs that can translate proteins or peptides.

Coding ncRNAs	Protein/Peptide	Cancer type	Expression	Mechanistic Theme(s)	Reference
Pri-miR-31	miPEP31	Foxp3+ regulatory T cells	down	transcriptional repressor	(39)
Pri-miR-34a	miPEP133	Nasopharyngeal carcinoma	up	Migration, invasion, apoptosis	(48)
LINC00665	CIP2A-BP	Breast cancer	down	Migration, invasion	(13)
LINC01234	MBOP	Colorectal cancer	up	ubiquitin–protease-system-directed degradation	(49)
LINC00998	SMIM30	Hepatocellular carcinoma	up	Cell proliferation, cell cycle, invasion, migration	(12)
LINC00961	SPAAR	Endothelial cell	up	angiogenesis	(50)
LINC00908	ASRPS	Triple-negative breast cancer	down	angiogenesis	(51)
LOC90024	SRSP	Colorectal cancer	up	Tumorigenesis, progression	(14)
HOXB-AS3	HOXB-AS3 peptide	Colon cancer	down	Metabolism reprogramming	(52)
LINC00266-1	RBRP	Cancers	up	Cell proliferation, invasion, migration, colony formation	(53)
LINC00467	ASAP	Colorectal cancer	up	Cell proliferation, mitochondrial metabolism	(54)
CTD-2256P15.2	PACMP	Cancers	up	tumor growth, sensitivity of tumors to radiotherapy and multiple targeted therapeutic agents	(55)
CircPINT	PINT87aa	Glioblastoma	down	Cell proliferation, neuro-sphere foemation, invasion ability	(56)
Circ-EIF6	EIF6-224aa	Triple-negative breast cancer	up	Ubiquitin-proteasome pathway	(57)
Circ-AKT3	AKT3-174aa	glioblastoma	down	Tumorigenesis, cell proliferation, radiation resistance	(58)
Circ-FBXW7	FBXW7-185aa	glioblastoma	down	Proliferation, cell cycle	(46)
CircGpr5a	Gprc5a	Bladder cancer stem cell	up	Tumorigenesis, metastasis	(59)
circPPP1R12A	circPPP1R12A-73aa	Colon cancer	up	Proliferation, migration, invasion	(47)
circβ-catenin	β-catenin	Liver cancer	up	Proliferation, migration	(60)
Circ-SHPRH	SHPRH-146aa	glioblastoma	down	Proliferation, tumorigenicity	(61)

## 4 Exosomal ncRNAs and cancer

**Figure 2:**
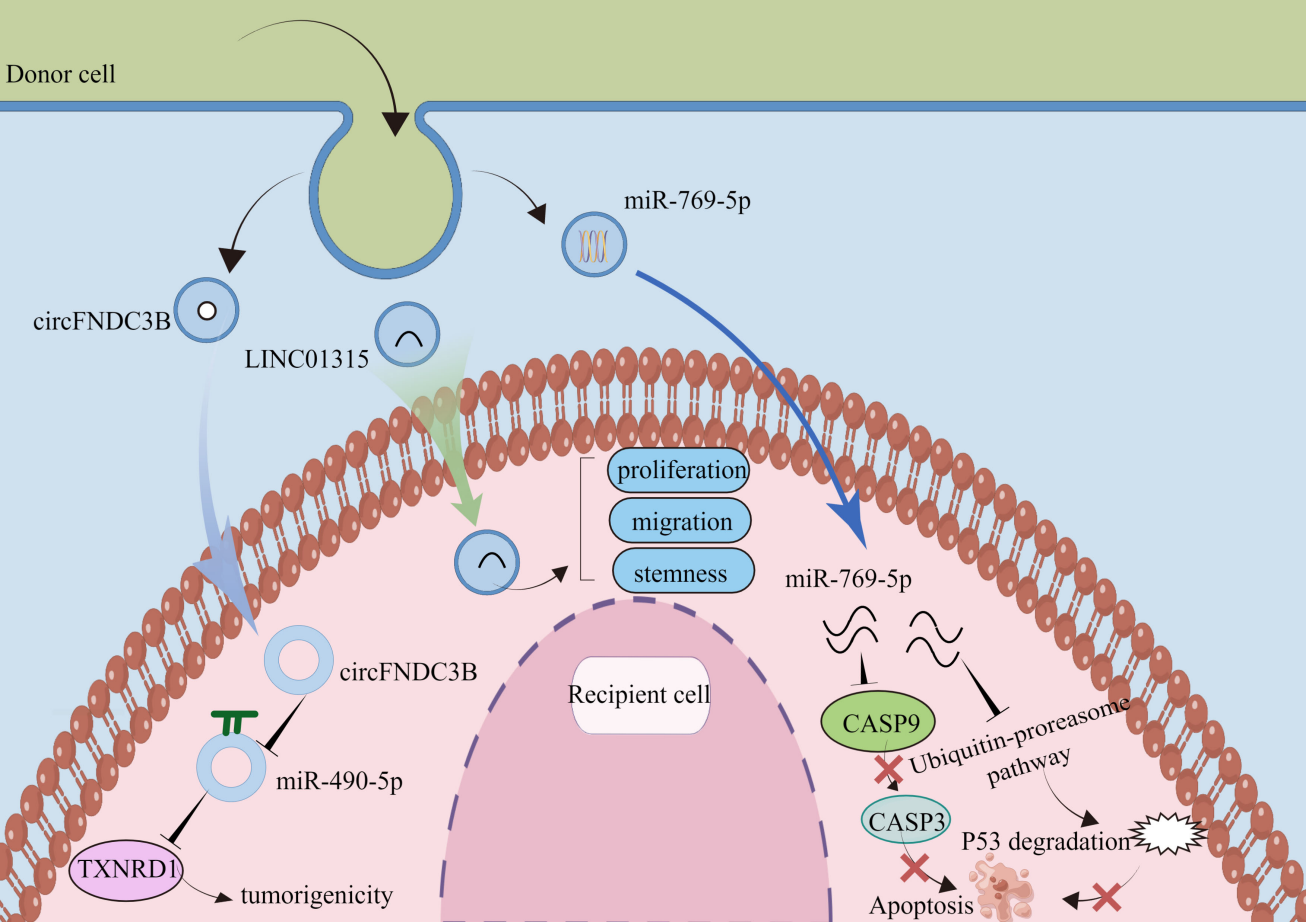
Tumor-derived exosomal ncRNAs function. (By Figdraw).

Exosomes are bilayer membrane vesicles of size 30-150nm, which are secreted to the extracellular uptake of recipient cells after serosal fusion, and play an important role in intercellular communication. Exosomes provide the recipient cells with multiple biomolecules, including lipids, proteins, DNA, and ncRNAs, mediating cell activation, phenotypic modification, and reprogramming of cellular functions. Working as a novel mode of intercellular communication, we summarize some of the mechanisms of action of exosome-carrying ncRNAs in tumors (**Figure 2**).

### 4.1 Exosomal microRNA and cancer

In patients with ovarian cancer, tumor cells produce exosomes, which are particularly abundant in malignant exudates, such as ascites (62). Exosomal miRNAs can be secreted in various body fluids, such as serum, plasma, urine, and peritoneal fluid, thereby avoiding ribonuclease degradation (63). Exosomal miRNAs in the circulating blood have been reported in most tumors for early cancer screening, disease course monitoring, and outcome assessment. Serum miR-100 levels were significantly lower in glioma patients than in healthy controls. However, they were significantly higher after treatment. Multivariate analysis was performed to identify serum miR-100 as an independent prognostic indicator in glioma patients (64).

Moreover, in colorectal cancer, miR-182 and miR-20a were up-regulated in tissues and plasma compared to those in healthy individuals. The area under the receiver operating characteristic (ROC) for circulating miR-182 at the validation stage was 0.891 (95% CI, 0.821–0.961), and the Area Under Curve (AUC) for circulating miR-20a expression was 0.801 (95% CI, 0.695–0.906) (65). Tumor-derived exosomes contribute to the immune escape of tumor cells by negatively regulating effector T and NK cells (66). To investigate how exosomal miRNAs derived from pancreatic cancer suppress the mRNA expression in dendritic cells, Ding et al. (67) found that their derived exosomes induced immune tolerance in dendritic cells by inhibiting the expression of the target gene RFXAP *via* miR-212-3p, through exosome stimulation of dendritic cells in pancreatic cancer. Whether exosomes can carry pre-miRNA-encoded peptides to play a role in tumor development and how they achieve this requires further exploration.

### 4.2 Exosomal LncRNAs and cancer

The bilayer membrane structure of exosomes effectively prevents their contents from being removed or experiencing RNA damage or degradation, further extending the circulating half-life of lncRNAs and enhancing their biological activity (68). Current research indicates that lncRNAs are closely associated with tumor cell invasion, metastasis, and immune system regulation (69). The process of exosome secretion is a multi-step process. Yang et al. (70) found that genes associated with exosome secretion were enriched in the HOTAIR high expression group. HOTAIR controls the docking process by regulating the expression and localization of RAB35. Further, HOTAIR facilitated fusion to complete the final step of exosome secretion by influencing the co-localization of VAMP3 and SNAP23. Moreover, several studies have suggested that exosomal lncRNAs can modulate chemoresistance. The results obtained by Wang et al. (71) showed that HOTTIP is highly expressed in cisplatin-resistant gastric cancer cells, and further studies revealed that exosomal HOTTIP promotes cisplatin resistance by activating HMGA1 in gastric cancer cells. Considering that tumor cells use various intercellular communication mechanisms to adapt to the local microenvironment, manipulate the immune system, and promote metastasis, the involvement of peptide/proteins in intercellular communication between tumor and stromal cells in the local or distant microenvironment *via* the exosomal pathway should be investigated in depth.

### 4.3 Exosomal circRNA and cancer

CircRNAs are endogenous RNAs that are structurally stable and conserved in sequence, with no 5’-end cap or 3’-end poly(A) tail (72). CircRNAs are more consistently present in cells, body fluids, and tissues than linear RNA (73). Memczak et al. (74) first proposed that circRNAs could compete with other RNAs for miRNA binding and play a role in post-transcriptional processes, laying the foundation for circRNAs in studying biological functions. Certain circRNAs are highly expressed at a predominant concentration at one locus, suggesting a tissue-specific expression pattern of circRNA (75). CircRNAs are enriched in exosomes and exhibit good stability (76). High-throughput sequencing analysis of exosomal RNA revealed different species of circRNAs in exosomes and cell plasma, indicating that circRNAs are selective in their entry into exosomes (77). However, the mechanism through which circRNAs enter exosomes remains unclear. Exosomal circRNAs may be essential mediators of communication between tumor cells and their microenvironment and play an important role in tumor development.

In non-small cell lung cancer (NSCLC), circ-MEMO1 is highly expressed in serum exosomes. Circ-MEMO1 promotes proliferation, cell cycle progression, and glycolysis in NSCLC cells by regulating the miR-101-3p/KRAS axis. High circ-MEMO1 expression predicts poor prognosis for patients with NSCLC. Additionally, it may be a valuable diagnostic marker for NSCLC (78). At the same time, circ-0051443 is expressed at low levels in the tissue and plasma exosomes of patients with hepatocellular carcinoma. Circ-0051443 is transported from normal cells to hepatocellular carcinoma cells by exosomes and inhibits the malignant biological behavior of hepatocellular carcinoma cells by affecting the cell cycle and apoptosis (79). Although a growing number of studies have shown the potential of circRNAs as tumor targets or diagnostic markers, further research is needed on the clinical applications of circRNAs. Exosomal circFNDC3B drove esophageal squamous cell carcinoma progression by miR-490-5p/TXNRD1 axis. Exosomal LINC01315 promotes proliferation, migration, and stemness. Exosomal miR-769-5p by the ubiquitin-proteasome pathway promotes degrading protein p53 (**Table 3**).

**Table 3:** ncRNAs in exosome.

Exosomal ncRNAs	Expression	Functional	Cancer Type	Reference
miR-455-3p	down	Cell apoptosis, autophagy	Myocardial cell	(80)
miR-21-5p	up	Proliferation, osteoblastic	Bone mesenchymal stem cell	(81)
miR-146a-5p	down	Macrophage polarization	cardiomyocyte	(82)
miR-26a-5p	down	Lymph node metastasis	Endometrial cancer	(83)
miR-769-5p	up	Cisplatin resistance, cancer progression	Gastric cancer	(84)
miR-423-3p	down	Cell progression, macrophage M2 polarization	Cervical cancer	(85)
miR-4466	up	Stemness, metabolic switch	Lung cancer	(86)
SND1-IT1	up	Malignant transformation	Gastric cancer	(87)
LncRNA ZFAS1	up	Proliferation, migration, invasion	Cervical cancer	(88)
LINC00152	up	Potential diagnosis biomarker	Gastric cancer	(89)
LncRNA RP5-977B1	up	Diagnostic and prognostic biomarker	Lung cancer	(90)
HOTAIR	up	Proliferation, migration, invasion	endometriosis	(17)
TUG1	up	migration, invasion, glycolysis	Cancer-associated fibroblasts	(91)
LINC01315	up	proliferation, migration, and stemness of cells	Colorectal cancer	(92)
CircVMP1	up	Proliferation, migration, invasion, apoptosis	Non-small cell lung cancer	(93)
Circ_0065149	down	Screening biomarker	Gastric cancer	(94)
CircDNER	up	Proliferation, invasion, migration	Lung cancer	(95)
CircRAPGEF5	up	Proliferation, migration, invasion	Lung adenocarcinoma	(96)
CircPLK1	up	migration, invasion, apoptotic	Non-small cell lung cancer	(97)
CircFNDC3B	up	colony formation, proliferation, migration, invasion, glycolysis	esophageal cancer	(98)

## 5 Relationship between peptide encoding by ncRNAs and exosome

Although the functions of peptide or proteins have been elucidated in liver, breast, and colorectal cancers, their mode of action remains incompletely understood. In light of recent studies suggesting that exosomes represent a new mode of intercellular communication, it is possible that peptide/proteins together with exosomes are involved in intercellular communication in the local or distant microenvironment. Cai et al. (99) identified 84 peptides in glioma cells and 29 peptides in their exosomes by improving the workflow and criteria for peptide-protein analysis, nine of which were present in both cells and exosomes. These results suggested that the release of specific peptides in or with exosomes may be actively regulated. Notably, tumor cells secrete exosomes that act as a bridge between tumor cells and their microenvironment. Thus, the derived exosomal peptides have the potential to regulate receptor cell function. In addition, exosomes contain a lipid bilayer membrane structure that prevents degradation of the nucleic acids and proteins they encapsulate, while their small size and membrane structure facilitate their uptake by tumor cells. The exosome peptides will therefore have greater function and integrity and great potential as diagnostic and prognostic markers (100).

## 6 Conclusion

This review summarizes many studies in which ncRNAs can encode peptides and proteins, in which ncRNAs carried by exosomes can act as biomarkers in tumors. Most importantly, the peptides encoded by ncRNAs in exosomes can serve as potential diagnostic markers, also as targets for therapeutic oncology agents. In the past time, peptides have been widely used in medicine. Currently, there are many peptide drugs in preclinical and clinical trials. Peptide drugs have the characteristics of high efficiency, safety and more tolerance, Furthermore, have the advantages of high selectivity and not easy to accumulate in the body. Although the current development of peptide drugs still faces great challenges, we believe that with the development of technology, the shortcomings of peptide drugs will eventually be overcome and the application scope of polypeptide drugs will be expanded. The limitations of this research field should to be point. Our current technology still cannot overcome the discovery of peptides such as low molecular weight and low abundance. Other ncRNAs such as snRNAs, tRNAs and rRNAs Is it possible to encode a new peptide/protein. What function ncRNAs binds to the ribosome but without an ORF remains unclear.

However, in future, several questions remain our to address. For example, what are the molecular mechanisms by which exosome peptides act, and how can the discovered molecular markers be translated into clinical use? Nevertheless, peptides in exosomes have the potential to play a key role in the diagnosis and treatment of tumors.

## Author contributions

Conceptualization, JY, ML and XF. Investigation, HZ and QR. Data curation, YZ. Writing—original draft preparation, JY. Writing—review and editing, YW and YNZ. All authors contributed to the article and approved the submitted version.
