# Supplementary figures and images for: Advances in peptides encoded by non-coding RNAs: A cargo in exosome
Source: Front Oncol. 2022 Dec 23;12:1081997. doi: 10.3389/fonc.2022.1081997 (PMC9822543; doi:10.3389/fonc.2022.1081997)

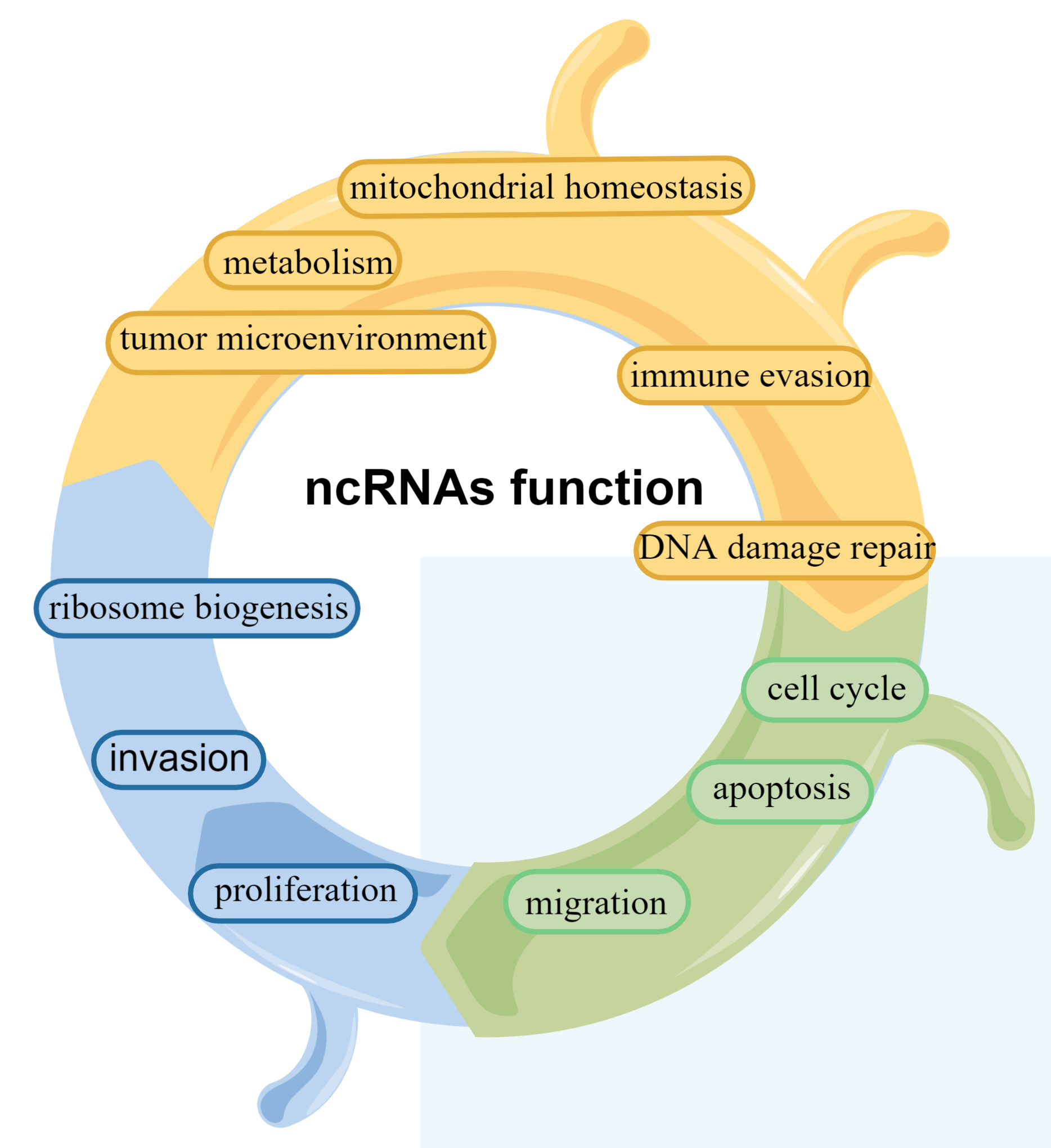

Supplement: Supplementary file 1 [file Image_1.tif]
